# Propagation Characteristics of Multi-Frequency Arc-Shaped Flat-Plate Ultrasound in Xanthan Gum Viscous Systems and Its Influence on Rheological Properties

**DOI:** 10.3390/foods14244226

**Published:** 2025-12-09

**Authors:** Lei Zhang, Haiyang Zhang, Ruonan Wang, Yujing Yan, Wenqi Zheng, Yan Shen, Xiaoyu Chai, Hafida Wahia, Chenglin Li, Zhenyuan Hu, Haile Ma, Cunshan Zhou

**Affiliations:** 1 School of Food and Biological Engineering, Jiangsu University, Zhenjiang 212013, China; 13861776963@163.com (H.Z.); wrn15132382003@163.com (R.W.); 3230910070@stmail.ujs.edu.cn (Y.Y.); zwq1225000000@163.com (W.Z.); liand1214@163.com (Y.S.); hafida.wahia.iaa@gmail.com (H.W.); lin3200910098@163.com (C.L.); huzhenyuan45@gmail.com (Z.H.); mhl@ujs.edu.cn (H.M.); 2 Institute of Food Physical Processing, Jiangsu University, Zhenjiang 212013, China; 3 Key Laboratory for Theory and Technology of Intelligent Agricultural Machinery and Equipment of Jiangsu University, Jiangsu University, Zhenjiang 212013, China; xfpaxy@ujs.edu.cn; 4 Jiangsu Province and Education Ministry Co-Sponsored Synergistic Innovation Center of Modern Agricultural Equipment, Jiangsu University, Zhenjiang 212013, China

**Keywords:** ultrasound, propagation characteristics, xanthan gum, rheological property

## Abstract

The solubility and rheological properties of high-molecular-weight xanthan gum (XG) are crucial to its functional performance and determine its applications. Ultrasound modifies these properties mainly by altering acoustic propagation in viscous systems, which depends strongly on concentration and frequency mode. In this work, the propagation behavior of three frequency modes (33 kHz mono-frequency, 20–40 kHz dual-frequency, and 20–50–68 kHz triple-frequency) of arc-shaped flat-plate ultrasound was systematically investigated in XG solutions, as well as their effects on solubility and rheological properties. Results showed that multi-frequency ultrasound generated stronger and more uniform acoustic fields, maintaining higher space peak temporal peak acoustic intensity (I_SPTP_) over a wide concentration range, which was superior to the significant attenuation observed in mono-frequency mode above 10.0 g·L^−1^. Ultrasonic treatment effectively increased solubility from 62.0 to 63.5% (untreated) to a maximum of 85.6% in the 20–40 kHz group. In terms of rheology, ultrasound reduced viscosity and altered viscoelastic behavior by disrupting the molecular network, with multi-frequency modes showing greater effects at higher concentrations. Surface tension decreased to a minimum of 58.4 mN·m^−1^ under mono-frequency treatment. Frequency sweep and creep recovery tests indicated enhanced chain mobility and improved structural recovery after ultrasound. Microstructure analysis confirmed fiber fragmentation and the formation of a microporous structure, especially under multi-frequency modes. Overall, the key mechanism lies in the ability of multi-frequency ultrasound to maintain effective acoustic propagation in viscous media, thereby enhancing solubility and modulating rheological behavior.

## 1. Introduction

Polysaccharides, one of the four fundamental biomacromolecules in living organisms, are formed by the linkage of multiple monosaccharides through glycosidic bonds. They are widely distributed in plants, animals, and microorganisms, can also be obtained via artificial synthesis, and serve as crucial bioactive substances with diverse functionalities, including antioxidant, antimicrobial, anti-inflammatory, immunomodulatory, and antitumor effects [1], which are extensively applied in food, pharmaceutical, chemical engineering, and cosmetic industries [2,3]. Xanthan gum (XG), a macromolecule polysaccharide, contains abundant hydroxyl groups that readily form hydrogen bonds with water, conferring excellent hydrophilicity. It dissolves in water to form a hydrophilic colloid, as a thickener to modify rheological properties of foods [4], and exhibits remarkable thickening capacity [5] and stability [6]. However, its relatively poor solubility restricts efficient utilization, and the rational modification provides a critical approach to broadening its applications [7].

Current modification methods for polysaccharides primarily include enzymatic [8], chemical, and physical approaches [9,10]. However, physical methods offer distinct advantages, such as low cost, environmental friendliness, operational simplicity, controllable reaction conditions and high efficiency. Notably, ultrasonic technology not only enhances the extraction efficiency of plant and microbial polysaccharides but can also be synergistically combined with other techniques (e.g., enzymatic treatment, plasma-activated water, and microwave-assisted extraction) to reduce molecular weight (MW) and improve solubility [11,12,13,14,15]. These findings demonstrate significant modification effects of ultrasound on polysaccharides from various sources. Studies have revealed that ultrasonic microjets, generated during mechanical bond rupture, induced intense and rapid mechanical responses in polysaccharides [16,17]. This process cleaves chemical bonds in the polysaccharide backbone, leading to controlled degradation [18]. Additionally, cavitation effects not only generate localized high-temperature and high-pressure conditions that disrupt polysaccharide bonds, but also produce free radicals that further contribute to polysaccharide degradation [19,20]. The modification of XG represents an effective strategy for enhancing its functional properties. The introduction of functional groups can significantly alter physicochemical characteristics, including solubility, viscosity, thermal stability, and antioxidant activity [16,21,22]. Ultrasonically modified XG demonstrates reduced shear-rate-dependent viscosity variation relative to its native counterpart, maintaining its non-Newtonian pseudoplastic behavior and exhibiting improved rheological properties. The structural and rheological properties of XG undergo significant modifications following ultrasonic treatment, with its MW decreasing substantially from 3.0 × 10^7^ Da to 1.4 × 10^6^ Da, and these changes are accompanied by alterations in both monosaccharide composition and the linkage patterns of sugar residues [23]. As a result, ultrasonic modification facilitates precise control over XG ‘s solubility and rheological properties, enabling the tailoring of its functional characteristics.

Physical and thermodynamic properties of substances can be changed after mixing, with alterations in volume further affecting the polymer’s energy and molecular orientation [24]. In high-molecular-weight polysaccharide solutions, hydrogen bonding, hydrophobic interactions, and intermolecular entanglements enhance intermolecular forces, thereby modifying solubility, stability and bioactivity [25]. By measuring properties such as viscosity and density for viscous system, studies have evaluated adiabatic compressibility, intermolecular free path length, acoustic impedance, ultrasonic attenuation coefficient, and relaxation time, contributing to a deeper understanding of the liquid’s intermolecular behavior [26].

Recent studies have highlighted several inherent limitations of mono-frequency ultrasound, which may restrict its effectiveness in modifying food biopolymers and complex matrices. First, mono-frequency systems typically generate a relatively uniform and steady-state acoustic field, resulting in cavitation events with lower intensity and limited spatial diversity, thereby reducing their ability to induce extensive structural modifications in macromolecules such as proteins and polysaccharides [27]. In addition, mono-frequency ultrasound often exhibits restricted penetration depth and uneven energy distribution, leading to non-uniform treatment within the system, particularly in food matrices with high viscosity or complex rheological properties [28]. These limitations collectively suggest that mono-frequency ultrasound may be insufficient for achieving comprehensive physicochemical modifications. Furthermore, the operational parameters for multi-frequency ultrasound application have been more precisely delineated. For instance, in research focusing on the modification of frozen wheat dough, ultrasound was applied utilizing mono- (20 kHz), dual- (20 and 40 kHz), and triple- (20, 40, and 60 kHz) frequency configurations. The treatments were conducted for durations of 10, 20, and 30 min at a fixed power density of 45 W·L^−1^, with the temperature rigorously maintained between 2 °C and 8 °C. By employing multiple frequencies concurrently, the multi-frequency ultrasound technique induced more complex vibrational and thermal effects, thereby facilitating a broader modulation of the physicochemical properties of polysaccharides [29,30,31]. The synergistic effect of dual-frequency ultrasound transforms the ultrasonic field into a non-steady-state acoustic pressure field, generating more cavitation bubbles and resulting in stronger cavitation effects. In addition, multi-frequency ultrasound synergistically improves thawing quality and enhances protein stability, further demonstrating its superior capability to intensify cavitation effects [32]. In another study, an ultrasonic system incorporated six bottom-mounted 80 kHz transducers (power density 85.4 W·L^−1^) and an immersed 20 kHz horn transducer (power density 210 W·L^−1^) enhances cavitation effects [33]. Similarly, in emulsion treatment, multi-mode ultrasound (mono: 20, 40, 60 kHz; dual: 20–40, 40–60, 20–60 kHz; triple: 20–40–60 kHz) applied via divergent multi-frequency sonication for 30 min significantly enhanced the ultrasonic cavitation effect and promoted non-covalent interactions between proteins and polysaccharides [34].

This study systematically investigated the propagation behavior of multi-frequency ultrasound in XG viscous systems. This work elucidated the underlying mechanisms of ultrasonic wave propagation in a viscous medium and analyzed variation patterns of solubility and rheological properties, which can provide a foundation for improving modification efficiency and broadening potential applications of polysaccharides by virtue of cavitation effects.

## 2. Materials and Methods

### 2.1. Materials

XG (Model 9270), with a viscosity of 1200–1400 mPa·s at 1% concentration, was purchased from Zhongxuan Biochemical Co., Ltd. in Ordos City, China, with a production origin in the Inner Mongolia Autonomous Region and a centralized procurement location.

### 2.2. Methods of Sample Preparation and Ultrasonic Modification

XG (2.0–20.0 g) was dissolved in 1 L of deionized water to prepare solutions of varying concentrations. To ensure complete dissolution, particularly at higher concentrations, solutions were stirred for 20 min in a constant-temperature water bath at 55 °C with a rotational speed of 500 rpm. Preliminary viscosity measurements identified five representative concentration gradients (2.0, 4.5, 7.0, 10.0, 12.0 g·L^−1^) for viscous model systems. Tap water was used during the processing stage, as each experiment with arc-shaped flat-plate ultrasound consumed approximately 48 L per trial. Given the large volume and frequency of tests (15 different frequency combinations and numerous repetitions), the total water usage reached approximately 0.8 t. Furthermore, all solutions for viscosity screening and material property testing were made with deionized water. Three optimal frequency modes (33, 20–40, 20–50–68 kHz) were selected for application across varying concentration gradients, with the 33 kHz mode operating at a single frequency, the 20–40 kHz mode employing dual simultaneous frequencies, and the 20–50–68 kHz mode utilizing triple simultaneous frequencies. The ultrasonic treatment was performed with the temperature of the ultrasonic treatment chamber maintained at 25 °C, and all ultrasonic modes were maintained at a power of 6.25 W·L^−1^. For details on equipment and frequency selection, please refer to the Supplementary Materials. In addition, the duration of ultrasonic treatment was uniformly set to 1 h. Subsequent analyses examined solubility, viscosity variations, surface tension, rheological behavior and microstructural changes to clarify the propagation dynamics and modification mechanisms of arc-shaped flat-plate ultrasound.

### 2.3. Measurement of Propagation Characteristics of Arc-Shaped Flat-Plate Ultrasound

#### 2.3.1. Real-Time Monitoring Method of Acoustic Field in the Time–Frequency Domain

**Figure 1:**
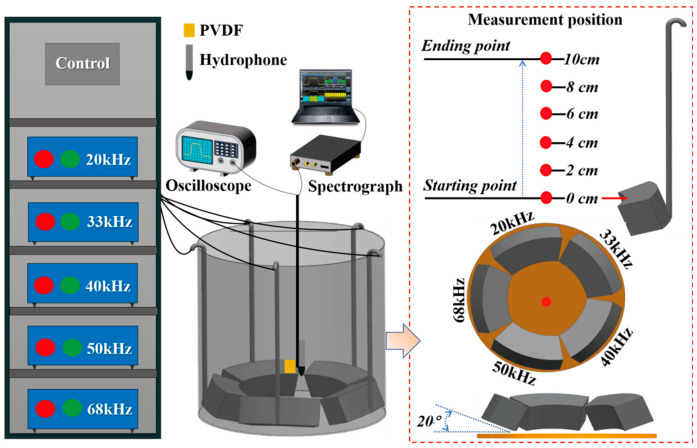
Multi-frequency arc-shaped flat-plate ultrasonic device with time–frequency domain monitoring system.

The real-time time–frequency monitoring system for ultrasonic field characterization was constructed during the modification of XG, as shown in Figure 1. The system comprised a polyvinylidene fluoride (PVDF) piezoelectric film sensor, an oscilloscope (Tektronix, Inc., Beaverton, OR, USA), an 8103 hydrophone (Bruel & Kjær, Virum, Denmark), and a spectrum analyzer (RSA306B, Tektronix, Inc., USA), which were employed for time-domain and frequency-domain signal acquisition and processing, with the former two dedicated to time-domain acquisition and the latter to frequency-domain processing, respectively.

The PVDF sensor captured time-domain acoustic signals by converting instantaneous acoustic pressure into electrical signals, which were subsequently displayed on the oscilloscope. The PVDF sensor featured an active area of 10 × 10 mm and a thickness of 30 μm, with a sensitivity of 2 × 10^−8^ V·Pa^−1^. A parallel-connected 50 Ω resistor ensured signal stability by minimizing noise interference. Simultaneously, the hydrophone monitored frequency-domain acoustic signals to determine the space peak temporal peak acoustic intensity (I_SPTP_) for quantitative ultrasonic field characterization. A real-time spectrum analyzer processed these signals to visualize the acoustic intensity distribution. The PVDF sensor and hydrophone were co-located, and the arc-shaped flat-plate ultrasonic transducer was oriented at a 20° emission angle.

Discrete measurement points were arranged, as illustrated in Figure 1, from 0 cm from the transducer surface at 2 cm vertical intervals (6 points total). This dual-domain monitoring system enabled comprehensive characterization of ultrasonic propagation dynamics, yielding reliable data for elucidating XG modification mechanisms while establishing a robust technical framework for process optimization and control.

#### 2.3.2. Normalization Analysis and Short-Time Fourier Transform (STFT)

Signal processing was conducted using normalization analysis and STFT for amplitude standardization and time–frequency characterization, respectively. Normalization analysis was applied to adjust signal amplitudes to a unified range with 0–1 or −1–1, eliminating variations caused by experimental conditions or equipment sensitivity. This process enhanced signal comparability and improved the stability of feature extraction [35]. STFT was implemented by dividing the signal into multiple short-time segments and performing a Fourier transform on each segment independently, thereby revealing localized frequency components and their temporal evolution.

The fundamental principle of STFT involves segmenting the signal using a windowing function, which overcomes the inherent limitation of the conventional Fourier transform in analyzing non-stationary signals with time-varying characteristics. In ultrasonic signal processing, STFT has been widely employed to investigate transient phenomena and characterize time–frequency properties of acoustic signals. In ultrasonic cavitation monitoring, STFT enables the detection of transient frequency variations associated with bubble dynamics (formation and collapse), thereby facilitating the quantitative assessment of cavitation intensity and its resultant material effects [36]. The mathematical formulation of STFT is expressed as (Equation (1)) [37]:

(1) $$X(t,\;f)\;=\;\int_{-\infty }^{\infty }x(\tau )\omega (\tau \;-\;t)e^{-j2\Pi f\tau }d\tau$$

where x(*τ*) represents the input signal, ω(*τ* − *t*) denotes the window function, *t* is time, and *f* is frequency.

### 2.4. Measurement of Rheological Properties of Xanthan Gum

#### 2.4.1. Solubility

The solubility measurement method was slightly modified from methods described in the literature [38,39]. Briefly, XG samples (both unmodified and ultrasonically modified) were freeze-dried in Petri dishes. Subsequently, 0.25 g of the dried XG was weighed into a 50 mL beaker, and 25 mL of distilled water was added. The pH of the sample was adjusted to 4 using a 0.1 mol·L^−1^ HCl solution. The mixture was stirred thoroughly and maintained at 40 °C for 30 min, followed by centrifugation at 4200 rpm at 25 °C for 20 min.

The supernatant was transferred to a beaker, evaporated to dryness in a 90 °C water bath, and then dried until reaching constant weight at 105 °C. The solubility (%) was calculated using Equation (2):

Solubility (%) = (m_1_/m_2_) × 100% (2)

where m_1_ is the mass of the dried supernatant (constant weight) and m_2_ is the mass of the original sample.

#### 2.4.2. Viscosity

Viscosity measurements were performed using a digital viscometer (NDJ-8S, Sannuo Instruments, Shenzhen) at 25 ± 1 °C for XG solutions before and after ultrasonic treatment. Triplicate measurements were conducted for each sample, with results expressed as mean values. The viscosity reduction ratio (V) was calculated according to Equation (3) [40]:

V = (η_1_ − η_2_)/η_1_ × 100% (3)

where η_1_ represents the initial viscosity and η_2_ denotes the post-modification viscosity.

#### 2.4.3. Surface Tension

Surface tension was measured at room temperature using the Du Nouy ring method with a tensiometer (QBZY series fully automatic surface tensiometer, Fangrui Instrument Co., Ltd., Shanghai, China). For air/water surface tension measurements, XG solutions before and after ultrasonic modification were prepared in triplicate, with three measurements performed for each solution. The surface tension values were calculated using Equation (4):

Γ(mN·m^−1^) = F/4Πr (4)

where F is the measured force and R is the radius of the platinum ring.

#### 2.4.4. Viscoelastic Property

The dynamic strain sweep tests were adapted from the literature with modifications [41]. Measurements were performed within a strain range of 0.01–1000% at a constant temperature of 25 °C and an angular frequency of 10 rad·s^−1^. The storage modulus (G′), loss modulus (G″), and stress and strain percentages within the linear viscoelastic region were recorded at their corresponding concentrations.

Following a previously reported small-amplitude frequency sweep method [42], with slight modifications, a strain of 50% was determined by dynamic strain sweep tests. Frequency sweeps were then performed from 0.1 to 100 rad·s^−1^ at 25 °C to record the storage (G′) and loss (G″) moduli as functions of angular frequency (ω).

Creep recovery tests were carried out to measure creep compliance, following a reported procedure [43]. All tests were conducted at a constant temperature of 25 °C using a parallel-plate geometry (40 mm diameter), with shear stresses determined from dynamic strain sweep measurements. The critical stresses for the XG solutions were established as follows: 0.1 Pa (2 g·L^−1^), 0.7 Pa (4.5 g·L^−1^), 0.7 Pa (7 g·L^−1^), 1.0 Pa (10 g·L^−1^), and 4.0 Pa (12 g·L^−1^). Each test comprised sequential 180 s creep and recovery phases.

Steady-state shear measurements were conducted using parallel-plate geometry (40 mm diameter) on a rheometer, with a cone angle of 0° and a fixed gap distance of 1000 μm. Prior to testing, all samples were equilibrated at 25 °C for 2 min to ensure thermal stability. To prevent solvent evaporation during measurements, silicone oil was carefully applied around the measurement gap.

### 2.5. Microstructure of Xanthan Gum

XG samples were lyophilized using a freeze dryer (Epsilon 2-6D LSC plus, Martin Christ Ltd., Osterode am Harz, Germany), and their surface morphology was characterized by Scanning electron microscope (SEM) (JSM-IT800, Japan Electronics Co., Tokyo, Japan) at magnification levels of 100×, 1000× and 2000×.

### 2.6. Data Processing and Analysis

Data analysis was performed using Microsoft Excel 2010 (Microsoft Corporation, Redmond, WA, USA) and OriginPro 8.0 (OriginLab Corporation, Northampton, MA, USA), with all measurements conducted in at least triplicate. Results are presented as mean ± standard deviation (SD). Statistical analysis was carried out using SPSS Statistics 23.0 (IBM Corporation, Chicago, IL, USA) with Duncan’s multiple range test, with statistical significance set at *p* < 0.05.

## 3. Results and Discussion

### 3.1. Study on Propagation Characteristics of Arc-Shaped Flat-Plate Ultrasound in Xanthan Gum Solutions with Different Viscosities

**Figure 2:**
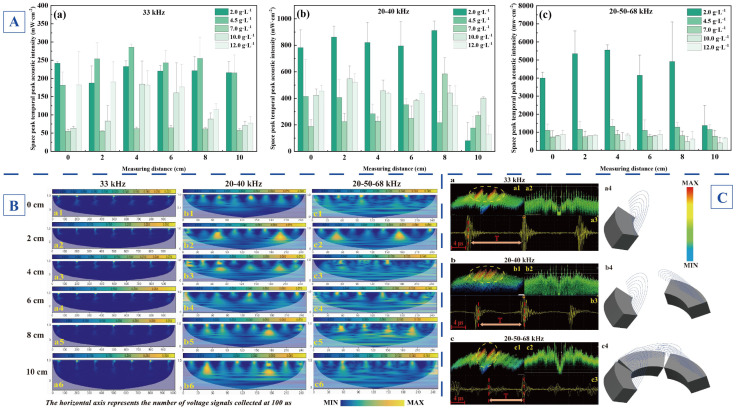
Study on propagation characteristics of arc-shaped flat-plate ultrasound in XG viscous systems (*p* < 0.05). (**A**) Spatial distribution of I_SPTP_ at monitored positions for three ultrasonic frequency modes (**a**: 33 kHz; **b**: 20–40 kHz; **c**: 20–50–68 kHz) in XG solutions (2.0, 4.5, 7.0, 10.0, 12.0 g·L^−1^). (**B**) Short-time Fourier transform (STFT) scalograms of three ultrasonic frequency modes (**a1**–**a6**: 33 kHz; **b1**–**b6**: 20–40 kHz; **c1**–**c6**: 20–50–68 kHz) at different monitoring positions of hydrophone and PVDF sensor relative to the ultrasonic transducer. (**C**) Time–frequency domain acoustic signal analysis of three ultrasonic frequency modes (**a**: 33 kHz; **b**: 20–40 kHz; **c**: 20–50–68 kHz) with (**a1**–**c1**) 3D spectral energy diagrams, (**a2**–**c2**) frequency-domain noise signal patterns, (**a3**–**c3**) time-domain noise signals, (**a4**–**c4**) schematic diagrams of transducer mechanisms.

Figure 2A displays the distribution of I_SPTP_ with different frequency modes in XG solutions at varying concentrations, revealing the complex relationship between ultrasonic intensity and ultrasonic propagation distance. The propagation characteristics of ultrasound in viscous solutions are closely related to the rheological behavior and microstructure of the medium. The XG, as an anionic polysaccharide colloid, exhibits concentration-dependent variations in solution viscoelasticity that significantly affects acoustic energy transfer mechanisms. As shown in Figure 2A(a), the 33 kHz mono-frequency ultrasound exhibits relatively balanced propagation characteristics at low concentrations (2.0, 4.5, 7.0 g·L^−1^), with minor variations in I_SPTP_, consistent with the strong penetration capability and homogeneous acoustic field characteristics of low-frequency modes. However, as the concentration increased to 10.0 g·L^−1^ and 12.0 g·L^−1^, significant differences in I_SPTP_ emerged across monitoring positions, showing progressive attenuation with distance until the I_SPTP_ dropped below 100 mW·cm^−2^ at 10 cm from the transducer surface.

Frequency superposition effectively enhanced acoustic field energy and improved energy distribution uniformity. Specifically, the I_SPTP_ under the 20–40 kHz dual-frequency ultrasonic mode increased threefold compared to that under mono-frequency mode, maintaining high energy levels within 8 cm from the transducer surface across all XG concentrations. The acoustic energy attenuates to its minimum at 10 cm position as shown in Figure 2A(b). Figure 2A(c) shows that the 20–50–68 kHz tri-frequency ultrasonic mode resulted in a significant enhancement of acoustic energy at the concentration of 2.0 g·L^−1^, with a maximum increase of up to 30 times within 0–8 cm of the transducer surface and about 6 times at the 10 cm position, compared to the mono mode. Though this enhancement becomes less pronounced at the other four high concentrations, the tri-frequency mode still maintained satisfactory spatial energy distribution throughout the propagation field.

Figure 2B employs STFT time–frequency analysis to further investigate ultrasonic propagation characteristics of three frequency modes (33, 20–40, 20–50–68 kHz) of arc-shaped flat-plate ultrasound. Although mono-frequency ultrasound mechanisms predominantly have been focused, multi-frequency synergistic effects and dynamic responses should be addressed more sufficiently. While it has been suggested that multi-frequency combinations enhance cavitation intensity via resonance effects, their impacts on XG at the molecular scale remain unclear. Moreover, conventional characterization methods, including viscosity measurement, inadequately capture transient dynamic behaviors during ultrasonic processing [44,45]. To address these gaps, STFT time–frequency analysis was used to systematically examine dynamic response characteristics during multi-frequency ultrasound treatment (33, 20–40, 20–50–68 kHz) of 4.5 g·L^−1^ XG solution. High-resolution scalograms elucidate mechanisms of multi-frequency synergy on XG’s molecular structure by revealing ultrasonic cavitation bubble dynamics and spatial distribution patterns across frequency bands. These scalograms, obtained via STFT, revealed dynamic frequency-response characteristics of multi-frequency ultrasound in XG solutions, where the normalized vertical axis coupled with time-domain voltage signal distribution provided critical experimental evidence for understanding interactions between ultrasonic cavitation effects and XG’s rheological properties. Signal variations across locations reflected transient cavitation bubble dynamics induced by ultrasound [46], with signal differences arising from multi-frequency ultrasonic synergy, which was correlated closely with either shear-thinning behavior or structural reorganization of XG molecules. Particularly, the broadband 20–50–68 kHz tri-frequency combination excited multiple resonance frequencies, enhancing cavitation intensity while optimizing energy transfer efficiency, offering practical guidance for industrial viscosity control of XG solutions [44].

Furthermore, the high-resolution capability of STFT time–frequency analysis enables precise detection of ultrasound-induced micro-scale flow field variations, establishing a data foundation for modeling cavitation intensity–polysaccharide degradation kinetics [46]. All three frequency modes (mono-, dual- and tri-frequency ultrasound) employed identical 100 μs acquisition durations. The 33 kHz mono-frequency scalograms in Figure 2B(a1–a6) show a relatively uniform signal amplitude distribution within 10 cm, with about seven pulse signals per acquisition period indicating periodic cavitation bubble generation and collapse under mono-frequency excitation, exhibiting high energy magnitude yet low energy density that induced localized shear-thinning without significant molecular restructuring. The 20–40 kHz dual-frequency ultrasound in Figure 2B(b1–b6) displays periodically modulated pulses with enhanced dispersion and marginally increased pulse density. The dual-frequency synergy expands the cavitation bubble distribution via superimposed resonance effects and enhances microjet impact forces, thereby potentially facilitating the targeted scission of XG molecular chains. In Figure 2B(c1–c6), the 20–50–68 kHz tri-frequency ultrasound combination exhibits sustained oscillatory superposition of transient energy peaks in scalograms, establishing a multi-peak resonance mode. This excitation mode significantly increases cavitation bubble generation range, achieves higher amplitude, and elevates energy density. Multi-frequency ultrasound thus enables optimized cavitation intensity and energy distribution through frequency synergy effects, demonstrating potential for precisely controlling XG’s chain scission and structural reorganization processes.

Figure 2C employs time–frequency domain ultrasonic signal monitoring to systematically analyze the operational mechanisms of three frequency modes (33, 20–40, 20–50–68 kHz) of arc-shaped flat-plate ultrasound. Ultrasonically generated noise signals interacted with surfaces of both the PVDF sensor and hydrophone probe. The PVDF sensor converted detected noise signals into electrical signals by oscilloscope display, while the hydrophone transmitted signals by spectrum analyzers, with acoustic intensity and noise signal strength quantified as voltage in the time domain and graphically visualized in the frequency domain. In Figure 2C(a1–c1), a real-time spectrum analyzer is used to generate 3D waterfall acoustic spectra from ultrasonic signals, and the color scale represents acoustic energy density, with red indicating regions of peak energy (corresponding to the most intense cavitation activity) and blue signifying low energy levels (associated with weak cavitation effects). Figure 2C(a2–c2) displays DPX fluorescence spectra, representing 2D front views of 3D waterfall plots, clearly showing variations in peak intensity and quantity across different ultrasonic frequencies. The oscilloscope’s voltage output exhibits direct proportionality to peak acoustic intensity in the ultrasonic field. Higher voltage amplitudes correlate with increased I_SPTP_ and more pronounced ultrasonic effects. Cavitation undergoes growth-to-collapse cycles driven by acoustic pressure variations. Distinct signal periods characterize different ultrasonic modes in Figure 2C(a3–c3), with multi-frequency ultrasound showing shorter periods that promote more rapid cavitation initiation compared to mono-frequency operation. Figure 2C(a4–c4) schematically illustrates operational mechanisms of ultrasonic modes, showing the propagation superposition of multi-frequency ultrasonic waves.

As a pseudoplastic colloid, XG’s rheological behavior was fundamentally governed by molecular chain entanglements and hydrogen bonding interactions. The arc-shaped flat-plate ultrasonic transducer improved spatial acoustic field distribution through multi-frequency synergy owing to its directional effects. The observed viscosity reduction at high concentrations is attributed to the ultrasound-induced disruption of the dense polysaccharide network, a conclusion further supported by subsequent structural analysis revealing compromised integrity and micropore formation . This study next focused on the mechanisms of multi-scale rheological properties by frequency modes (mono- and multi-frequency) of arc-shaped flat-plate ultrasound.

### 3.2. Effect of Arc-Shaped Flat-Plate Ultrasound on Rheological Properties of Xanthan Gum Viscous Systems

#### 3.2.1. Solubility

**Figure 3:**
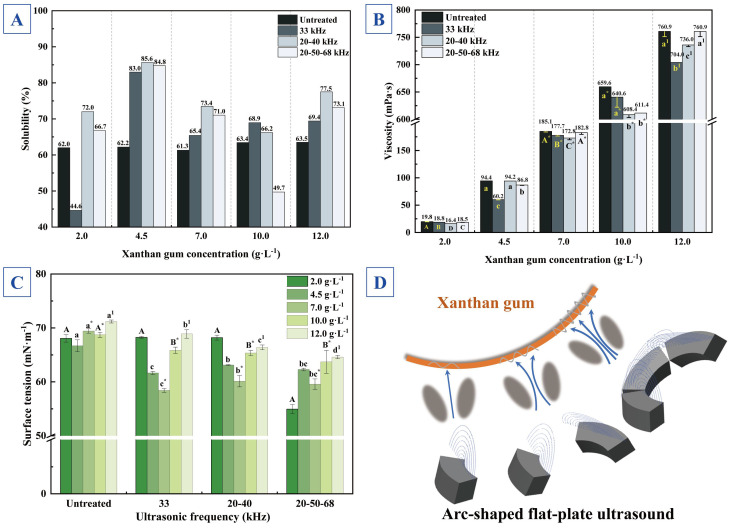
Effect of arc-shaped flat-plate ultrasound on interface properties of XG viscous systems (*p* < 0.05). (**A**) Solubility (data for 4.5 g·L^−1^ cited from Figure S2A), (**B**) viscosity (data for 4.5 g·L^−1^ cited from Figure S2B), (**C**) surface tension (statistical significance is indicated by different groups: uppercase letters, lowercase letters, lowercase letters with superscripts, and uppercase letters with superscripts), (**D**) schematic diagrams of ultrasonic interface mechanisms.

Figure 3A reveals that XG solutions (2.0, 4.5, 7.0, 10.0, 12.0 g·L^−1^) treated with three ultrasonic frequency modes (33 kHz mono-frequency, 20–40 kHz dual-frequency, and 20–50–68 kHz tri-frequency) exhibited sustainably enhanced solubility compared to untreated samples, with solubility increasing from 62.0 to 63.5% (untreated) to a maximum of 85.6% (20–40 kHz dual-frequency ultrasound-treated). Mono-frequency ultrasound showed inferior solubility enhancement compared to multi-frequency modes. Generally, ultrasound treatment increased solubility in most cases, attributable to mechanical effects of acoustic cavitation that disrupted XG’s side chains and glycosidic bonds in the backbone, with structural modifications promoting dissolution [23,47]. Furthermore, it has been suggested that a reduction in the hydrodynamic radius of high-molecular-weight colloids enhances solubility by improving molecular mobility and decreasing entanglements [48].

#### 3.2.2. Viscosity

Figure 3B illustrates effects of ultrasonic modification on viscosity in various XG viscous systems. Under 33 kHz mono-frequency ultrasound, all XG solutions exhibited viscosity reduction to varying degrees, with the maximum viscosity reduction ratio of 36.2% at 4.5 g·L^−1^ concentration. Increasing concentration shortened intermolecular distances, enhancing interaction forces and decreasing acoustic energy transfer efficiency, which aligned with the acoustic shielding theory in viscous systems [49]. The 20–40 kHz dual-frequency ultrasound induced maximum viscosity reduction of 17.2% at 2.0 g·L^−1^, primarily attributed to nonlinear cavitation bubble cluster resonance induced by secondary Bjerknes forces. The system was in the semi-dilute regime (c/c* ≈ 3, where c is polymer concentration and c* is critical overlap concentration), where acoustic pressure energy transferred effectively without reaching the strong acoustic attenuation threshold of concentrated solutions [50]. The 20–50–68 kHz tri-frequency ultrasound showed significant degradation effects at a concentration of 10.0 g·L^−1^. This was manifested in the formation of enlarged and more numerous micropores, which severely compromised the structural integrity and led to a pronounced viscosity drop, as revealed by subsequent microscopic analysis. In contrast, an induced viscosity increase was observed at 12.0 g·L^−1^. This phenomenon may have related to acoustic impedance matching conditions, that is, impedance mismatch may have caused sound wave reflection and refraction, while excessive acoustic intensity may have promoted molecular reorganization in colloidal systems [51].

#### 3.2.3. Surface Tension

Figure 3C demonstrates the effect of arc-shaped flat-plate ultrasound on surface tension in XG viscoelastic systems. The most significant surface tension reduction occurred under 33 kHz mono-frequency ultrasound treatment, decreasing from approximately 69.4 mN·m^−1^ to 58.4 mN·m^−1^ at 4.5 g·L^−1^ concentration. As a microbial-fermentation-derived polysaccharide, XG possesses a distinctive rod-like double-helical secondary structure with side chains winding inversely around the backbone, conferring high viscosity and molecular entanglement characteristics [52]. Mono-frequency ultrasound is known to induce helix unwinding and chain scission, reducing MW while exposing additional hydrophobic groups. The disruption of the native molecular architecture, as subsequently revealed by the observation of micropore formation and edge collapse, creates new surfaces and enhances hydrophobic group exposure at the molecular level. This structural modification resembled the ultrasound-induced hydrophobic region exposure mechanism in mung bean milk proteins [53], where increased hydrophobic exposure presumably reduced surface energy, consequently altering surface tension. In contrast, multi-frequency ultrasonic treatments (20–40, 20–50–68 kHz) showed attenuated effects, likely due to complex cavitation patterns and excessive acoustic intensity promoting molecular re-entanglement. Although Figure 2 indicates higher energy levels of I_SPTP_ during multi-frequency application, the I_SPTP_ drops sharply under viscosity constraints, suggesting modification of the semi-dilute regime under multi-frequency ultrasound. This is corroborated by the viscosity variations shown in Figure 3B. The concentration-dependent surface tension responses to different frequency modes, reflecting XG’s rheological state-dependent behavior. Solution parameters, such as viscoelastic moduli and relaxation spectra, varied with concentration, which governed ultrasound response mechanisms (Figure 3D), warranting further rheological investigation.

#### 3.2.4. Viscoelasticity

(1) Frequency Sweep

**Figure 4:**
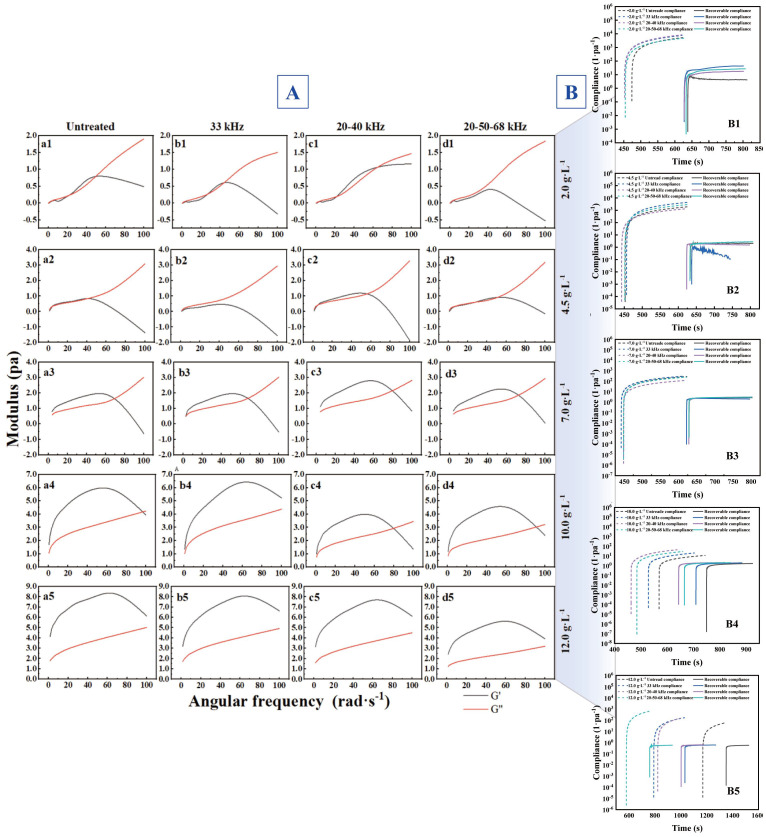
Effect of arc-shaped flat-plate ultrasound on viscoelasticity of XG viscous systems (*p* < 0.05). (**A**) Modulus for three ultrasonic frequency modes (**a**: untreated; **b**: 33 kHz; **c**: 20–40 kHz; **d**: 20–50–68 kHz) in XG solutions (2.0, 4.5, 7.0, 10.0, 12.0 g·L^−1^). (**B**) Compliance during creep and recovery phases in XG solutions (**B1**: 2.0; **B2**: 4.5; **B3**: 7.0; **B4**: 10.0; **B5**: 12.0 g·L^−1^).

Figure 4A shows variations in storage modulus (G′) and loss modulus (G″) of untreated and ultrasound-treated XG viscous solutions (30, 20–40, 20–50–68 kHz) over an angular frequency range of 0–100 rad·s^−1^. Rheological characteristics reflected viscous behavior alterations, revealing ultrasound-induced modifications in both rheological properties and molecular structure of XG solutions. It was demonstrated that ultrasound treatment could weaken shear-thinning behavior in pectin [54], consistent with previous reports showing ultrasound-induced structural adjustment of industrial hydrocolloids that can optimize mixing, solubility, and processing performance in food and pharmaceutical applications [55,56]. Results are presented in four panels (a–d) showing dynamic rheological properties of XG solutions at different concentrations. Generally, G′ increased initially, then decreased with rising angular frequency, while G″ monotonically increased with angular frequency. A viscoelastic transition zone appeared at low concentrations, which shifted upward with increasing concentration. However, this transition zone varied with different ultrasonic frequencies.

This resulted from the disruption of hydrogen bonds and other non-covalent interactions between XG molecules, along with weakened intramolecular interactions within polymer chains [57]. The initial behavior G′ > G″ at certain frequencies might have arisen from ultrasound-induced conformational transitions, due to compacted molecular structures [58], but shifted to viscous dominance (G″ > G′) at higher frequencies in dilute solutions. Results indicated concentration predominantly governed the viscoelastic regime of XG solutions, while ultrasound can modulate the transition point frequency-dependently. Solutions at 7.0 g·L^−1^ and 10.0 g·L^−1^ initially exhibited solid-like behavior, transitioning from elastic to viscous dominance with increasing frequency. The 12.0 g·L^−1^ solution maintained solid-like characteristics, though G′ and G″ showed converging trends at elevated frequencies. Different ultrasound frequencies induced distinct modification effects, with tri-frequency mode demonstrating optimal performance. As an eco-friendly technology, frequency-optimized ultrasound enhanced degradation efficiency while conserving energy, showing the potential value of ultrasound in viscosity regulation.

(2) Creep recovery

Figure 4B depicts the creep recovery behavior of XG solutions at various concentrations after ultrasound modification (33, 20–40, 20–50–68 kHz). Under constant stress conditions, strain increased with time. All samples exhibited nonlinear stress responses, representing time-dependent cumulative strain under constant stress. Where *J*(*t*) denotes creep compliance, *Jr*(*t*) recovery compliance, *γ*(*t*) and *γr*(*t*) are dimensionless shear strain parameters, and σ_0_ is the constant stress, as expressed by Equations (5) and (6):

*J* (*t*) = γ(*t*)/*σ*_0_ (5)

Jr(t) = γr(t)/σ_0_ (6)

During the creep phase, *J*(*t*) increased significantly with time, indicating progressive accumulation of viscoelastic deformation under constant stress. It was demonstrated that creep behavior correlated with molecular deformability and mobility in gel systems [59], and similar correlations have been reported in ultrasound-modified polysaccharides used in industrial suspensions, coatings, and personal-care gels [60]. XG systems treated at 33, 20–40 and 20–50–68 kHz exhibited minor compliance variations, though sharing similar trends, suggesting multi-frequency ultrasound affected material fluidity. During recovery phase, *Jr*(*t*) showed marked differences, and untreated samples showed 7 Pa^−1^ versus 50 Pa^−1^ for 33 kHz-treated solutions. Recovery compliance represented the ratio of residual strain to initial stress after load removal. Increased values indicate enhanced viscous dissipation [61]. Low-concentration samples displayed partial elastic recovery, yet ultrasound-treated groups exhibited significantly higher *Jr*(*t*) than controls, reflecting slower network reformation and weakened elasticity post-stress. Notably, 20–40 kHz dual-frequency treatment yielded maximum *Jr*(*t*), suggesting frequency-specific modulation of chain entanglement optimized energy dissipation pathways.

Ultrasonic modification significantly reduced the compliance of XG viscoelastic systems, with more pronounced effects observed under multi-frequency ultrasound treatment. Although compliance values varied slightly across frequencies, the overall trends were consistent, with multi-frequency modes demonstrating synergistic enhancement effects. indicating that frequency modulation could further optimize the elastic recovery performance.

### 3.3. Microstructural Analysis of Xanthan Gum

**Figure 5:**
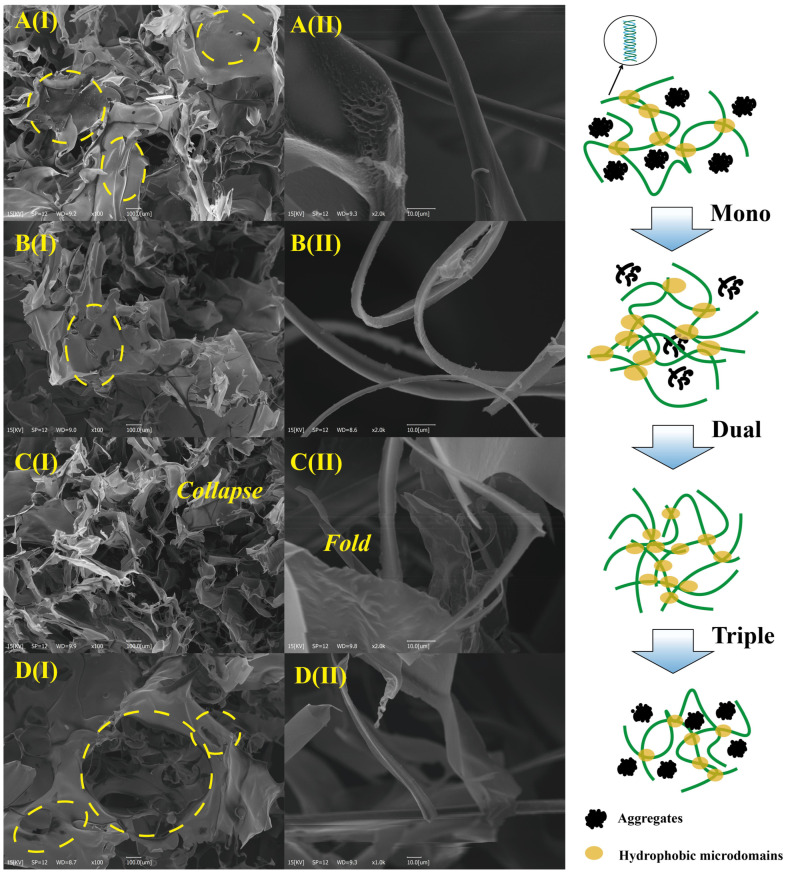
Microstructural morphologies of 4.5 g·L^−1^ XG treated by arc-shaped flat-plate ultrasonic frequency modes. (**A**) Untreated, (**B**) 33 kHz, (**C**) 20–40 kHz, (**D**) 20–50–68 kHz.

SEM is a highly effective technique for characterizing morphological features of macromolecules, including polysaccharides [62]. Figure 5 presents SEM micrographs of XG at 100×, 1000×, and 2000× magnifications. Figure 5A(I) shows intact surface morphology of untreated XG at 100×, while Figure 5A(II) reveals smooth and continuous fibrous structures at 2000×. Ultrasonic treatment induced micropores and edge collapse, compromising structural integrity, attributable to ultrasound-induced scission of glycosidic bonds [23,63]. Figure 5B(I),C(I) demonstrate micropore formation after 33 kHz mono-frequency and 20–40 kHz dual-frequency treatment, whereas Figure 5D(I) shows enlarged and more numerous micropores following 20–50–68 kHz tri-frequency treatment. Correlated with the data in Figure 3A (solubility) and Figure 3B (viscosity), multi-frequency ultrasound demonstrates superior modification efficacy due to synergistic effects.

## 4. Conclusions

Propagation behaviors of multi-frequency arc-shaped flat-plate ultrasound in XG viscous systems and its influence on rheological properties were analyzed based on experimental and numerical analysis. The research conclusions were as follows.

(1) Acoustic field propagation analysis revealed that frequency superposition effectively enhanced acoustic energy and improved its distribution uniformity. The I_SPTP_ of the 20–40 kHz dual-frequency ultrasound mode was significantly higher than that of the mono-frequency mode, and maintained high energy levels in all concentrations of XG solutions within 8 cm of the transducer surface. The 20–50–68 kHz tri-frequency ultrasound mode enhanced acoustic energy to a level much higher than that of the mono-frequency mode at low concentrations of 2.0 g·L^−1^, and maintained satisfactory spatial energy distribution at other higher concentrations.

(2) After treatment with the three ultrasonic frequency modes, the solubility of XG solutions was enhanced, with the highest increment from 62.0 to 63.5% in untreated samples to 85.6% in the 20–40 kHz dual-frequency ultrasound. The 33 kHz mono-frequency ultrasound achieved a maximum viscosity reduction of 36.2% at a concentration of 4.5 g·L^−1^. The multiple frequencies (20–40 kHz, 20–50–68 kHz) achieved significant viscosity reduction at 10 g·L^−1^, but a decrease in viscosity reduction ability was observed at a concentration of 12.0 g·L^−1^. The 33 kHz mono-frequency ultrasonic treatment showed the most significant effect at a concentration of 4.5 g·L^−1^, reducing the surface tension from about 69.4 mN·m^−1^ to 58.4 mN·m^−1^.

(3) Rheological frequency scanning and creep recovery tests further showed that ultrasonic treatment changed the frequency dependence of storage modulus (G′) and loss modulus (G″), and significantly improved the recovery compliance *J_r_*(t). In a 2.0 g·L^−1^ solution, *J_r_*(t) after 33 kHz treatment reached about 50 Pa^−1^, much higher than that of the untreated sample about 7 Pa^−1^. The microstructural damage was caused by ultrasound, especially the multi-frequency modes, to the XG structure, manifested as fiber structure fracture and micropore formation.

In conclusion, this study demonstrated that multi-frequency ultrasonic collaboration can overcome the energy limitations of mono-frequency systems by optimizing cavitation intensity and spatial distribution, and was expected to achieve precise molecular-scale modification. It provided theoretical support for the directional regulation of rheological properties of colloids in the food industry and a technical foundation for the development of efficient, low-energy consumption, and multi-frequency ultrasonic processing equipment. Future research should focus on clarifying the quantitative relationship between multi-frequency resonance and molecular chain dynamics, and establishing a generalized model of ultrasonic processing.

## Data Availability

The original contributions presented in this study are included in the article/Supplementary Material. Further inquiries can be directed to the corresponding authors.

## Supplementary Materials

The following supporting information can be downloaded at: https://www.mdpi.com/article/10.3390/foods14244226/s1, Figure S1: Influence of ultrasonic transducer configurations on solubility and viscosity of 4.5 g·L^−1^ XG solution (*p* < 0.05); Figure S2: Effects of arc-shaped flat-plate ultrasound with different frequency modes on XG solution (4.5 g·L^−1^). (A) Solubility, (B) viscosity. (Note: Data for 40 kHz cited from Figure S1 of Supplementary Materials.); Figure S3: Space peak temporal peak acoustic intensity (I_SPTP_) distribution monitored at discrete points in viscous system of XG (4.5 g·L^−1^) under 15 frequency modes of arc-shaped flat-plate ultrasound (Note: Data for 4.5 g·L^−1^ cited from Figure S2); Figure S4. Strain scan chart for 4.5 g·L^−1^ XG.
