# Supplementary material for: Propagation Characteristics of Multi-Frequency Arc-Shaped Flat-Plate Ultrasound in Xanthan Gum Viscous Systems and Its Influence on Rheological Properties
Source: Foods. 2025 Dec 9;14(24):4226. doi: 10.3390/foods14244226 (PMC12731867; doi:10.3390/foods14244226)
Supplement: Supplementary file 1 [file foods-14-04226-s001.zip › foods-4007670-supplementary (1).pdf]

Supplementary Materials

Propagation Characteristics of Multi-Frequency Arc-Shaped Flat-Plate Ultrasound in Xanthan Gum Viscous Systems and Its Influence on Rheological Properties

Lei Zhang [1,2,3,4,*,†], Haiyang Zhang [1,†], Ruonan Wang [1], Yujing Yan [1], Wenqi Zheng [1], Yan Shen [1], Xiaoyu Chai [3,4], Hafida Wahia [1], Chenglin Li [1], Zhenyuan Hu [1], Haile Ma [1,2] and Cunshan Zhou [1,2,*]

[1] School of Food and Biological Engineering, Jiangsu University, Zhenjiang 212013, China
[2] Institute of Food Physical Processing, Jiangsu University, Zhenjiang 212013, China
[3] Key Laboratory for Theory and Technology of Intelligent Agricultural Machinery and Equipment of Jiangsu University, Jiangsu University, Zhenjiang 212013, China
[4] Jiangsu Province and Education Ministry Co-Sponsored Synergistic Innovation Center of Modern Agricultural Equipment, Jiangsu University, Zhenjiang 212013, China
* Correspondence: zhangleifd@ujs.edu.cn (L.Z.); cunshanzhou@163.com (C.Z.); Tel.: +86-511-88780201; Fax: +86-511-88780201
† These authors contributed equally to this work.

1. Methods

1.1. Ultrasonic Modification Methodology Based on Types of Ultrasound Equipment

Xanthan gum solutions were formulated at a uniform concentration of 4.5 g·L^{-1} in tap water and were subsequently processed using four distinct ultrasonic apparatuses. The four ultrasonic devices are shown in Figure S1: ① A custom-made focused ultrasonic device (horn-type), ② a commercial ultrasonic bath (KQ-300DE, Kunshan Ultrasonic Instruments Co., Ltd., bath-type), ③ a self-made flat-plate ultrasonic device (bath-type), ④ a self-made arc-shaped flat-plate ultrasonic device (bath-type). All ultrasonic treatments were performed at a fixed frequency of 40 kHz and a power density of 6.25 W·L^{-1}, while the temperature was maintained constant at 25 °C. Furthermore, due to its directional emission characteristics, the arc-shaped flat-plate ultrasonic device was the primary focus of this study, enabling detailed investigation of the acoustic field distribution within the viscous system of the xanthan gum solution. The ultrasonic transducers were operated at five discrete frequencies (20, 33, 40, 50, and 68 kHz), generating 15 combined modes to treat the 4.5 g·L^{-1} xanthan gum solution. These modes included mono-frequencies and their combinations: 20, 33, 40, 50, 68, 20-33, 20-40, 20-50, 20-68, 20-33-40, 20-40-50, 20-50-68, 20-33-40-50, 20-40-56-68, and 20-33-40-50-68 kHz. The ultrasonic treatment was performed with all frequencies in each combination operating simultaneously, where mono-frequency modes utilized a single frequency, dual-frequency modes employed two simultaneous frequencies, and triple or higher-order modes utilized three or more simultaneous frequencies accordingly. The temperature of the ultrasonic treatment chamber was maintained at 25°C and the power density was maintained at 6.25 W·L^{-1}.

1.2. Solubility of Xanthan Gum

The solubility measurement method was slightly modified from methods described in the literature [1,2]. Briefly, xanthan gum samples (both unmodified and ultrasonically modified) were freeze-dried in Petri dishes. Subsequently, 0.25 g of the dried xanthan gum was weighed into a 50 mL beaker, and 25 mL of distilled water was added. The pH of the sample was adjusted to 4 using a 0.1 $mol{\cdot}L^{-1}$ HCl solution. The mixture was stirred thoroughly and maintained at 40 °C for 30 min, followed by centrifugation at 4200 rpm at 25 °C for 20 min.

The supernatant was transferred to a beaker, evaporated to dryness in a 90 °C water bath, and then dried until reaching a constant weight at 105 °C. The solubility (%) was calculated as Eq. S(1):

$$\text{Solubility (\%)} = (m_1/m_2) \times 100\% \qquad \text{S(1)}$$

where m_1 is the mass of the dried supernatant (constant weight), and m_2 is the mass of the original sample.

1.3. Viscosity of Xanthan Gum

Viscosity measurements were performed using a digital viscometer (NDJ-8S, Sannuo Instruments, Shenzhen) at 25±1 °C for xanthan gum solutions before and after ultrasonic treatment. Triplicate measurements were conducted for each sample, with results expressed as mean values. The viscosity reduction ratio (V) was calculated according to Eq. S(2)[3]:

$$V=(\eta_1-\eta_2)/\eta_1\times100\% \qquad \text{S(2)}$$

where η_1 represents the initial viscosity and η_2 denotes the post-modification viscosity.

1.4. Viscoelastic Property

The dynamic strain sweep tests were adapted from the literature with modifications[4]. Measurements were performed within a strain range of 0.01%-1000% at a constant temperature of 25 °C and an angular frequency of 10 $rad{\cdot}s^{-1}$. The storage

modulus (G'), loss modulus (G"), and stress and strain percentages within the linear viscoelastic region were recorded at their corresponding concentrations.

2. Results and Discussion

2.1. Effects of Multi-Mode Ultrasonic Transducer Configurations on Xanthan Gum Rheological Properties

Figure S1 illustrates the effects of different ultrasonic transducers on the solubility and viscosity of xanthan gum (4.5 g·L^{-1}). The solubility in the control group (non-sonicated) was 62.1%, and the maximum viscosity was 94.4 mPa·s, indicating the preservation of intact macromolecular polymer structures in solution. Compared to the control, all sonicated groups exhibited modified solubility and viscosity profiles. The focused ultrasound group significantly reduced viscosity to 43.7 mPa·s while marginally decreasing solubility to 61.5%. This modality concentrates acoustic energy within a small volume, generating intense cavitation effects that effectively cleave xanthan gum polymer chains and thereby substantially reduce viscosity. However, its localized focusing energy created limited cavitation zones, resulting in non-uniform solution modification.

The solubility in the ultrasonic bath group was 60.0% with a viscosity of 72.5 mPa·s for xanthan gum. The ultrasonic bath generated abundant microbubbles and spatially uniform cavitation, though the low intensity was insufficient to substantially enhance solubility or reduce viscosity. In contrast, the flat-plate ultrasound treatment reduced the solubility to 56.1% and resulted in a viscosity of 87.0 mPa·s. Conformational changes were induced wherein the decline in solubility correlated with denser molecular packing [5]. The arc-shaped flat-plate ultrasound group demonstrated optimal performance achieving a solubility of 64.9% and viscosity of 24.2 mPa·s. This improvement was attributed to its unique energy distribution, which creates multiple

high-intensity cavitation zones, thereby maximizing viscoelastic system resonance and molecular modification efficacy.

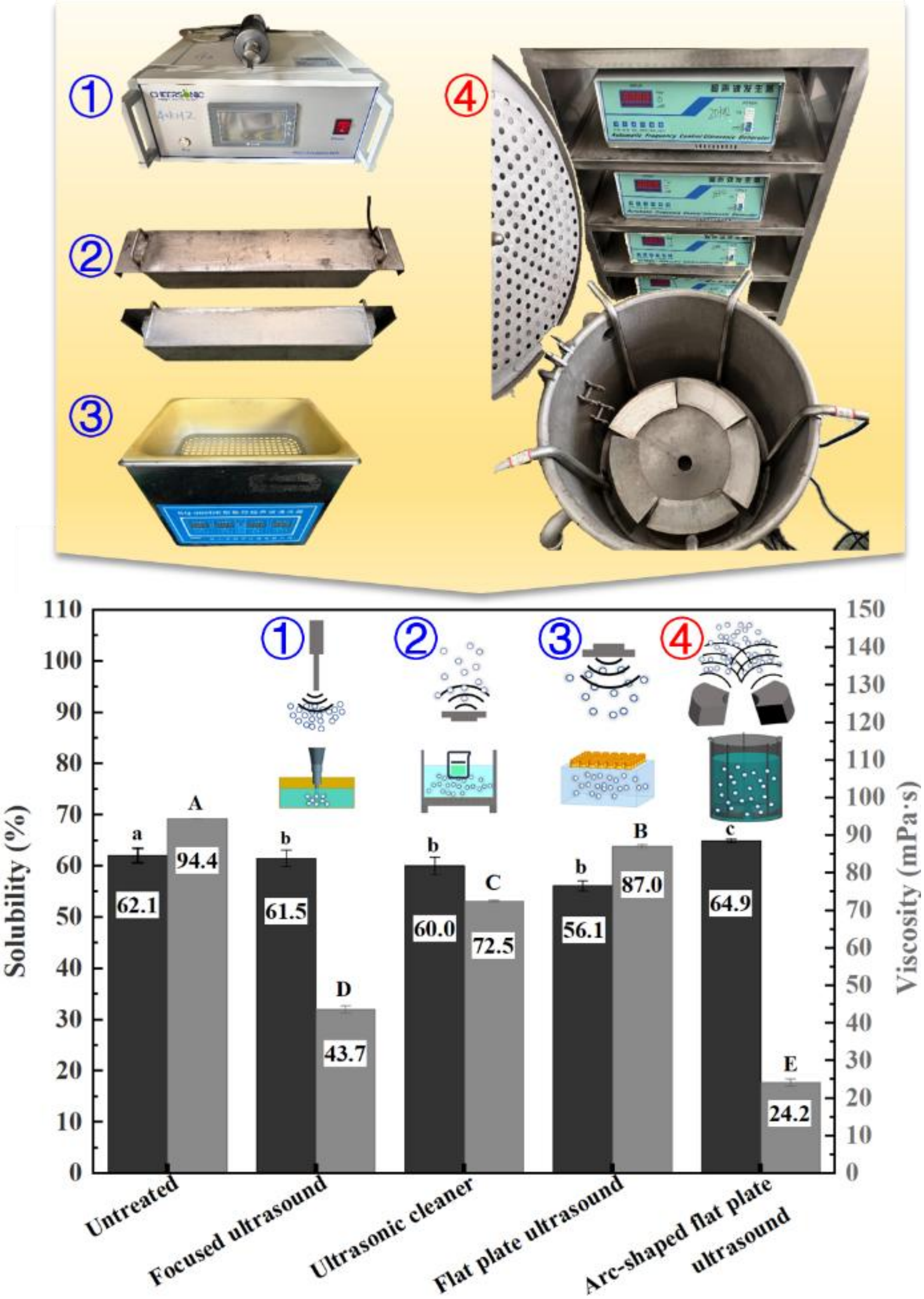

Figure S1. Influence of ultrasonic transducer configurations on solubility and viscosity of 4.5 g·L^{-1} XG solution (p<0.05).

The solubility and viscosity of xanthan gum are intrinsically linked to its molecular architecture, which consists of a pentasaccharide repeating unit (two glucose, two mannose, and one glucuronic acid) forming rigid helical structures [6]. Side-chain acetyl and pyruvate groups play critical roles; pyruvate moieties facilitate aqueous dispersion through their carboxylate anions, while acetyl groups promote hydrophobic interactions, forming viscous three-dimensional networks [7]. Structural modifications arise from cavitation-induced chain scission and alterations to these side groups.

Ultrasonic transducer configurations exhibit distinct modification effects on polysaccharides. For instance, both 20 kHz ultrasonic homogenizers with conical tips [8] and ultrasonic cell disruptors [9]—which fundamentally operate on focused-ultrasound principles with localized energy concentration—have been shown to effectively reduce the MW of mulberry branch polysaccharides across various power levels. Notably, cell disruptors can significantly decrease the MW of native polysaccharides without altering their fundamental structure, while simultaneously enhancing their radical (e.g., DPPH, ABTS, ·OH)-scavenging activity.

The ultrasonic bath (plate-type ultrasound) provided a wide and uniform energy distribution with 700 W/40 kHz treatment of *Polygonatum* polysaccharides [10]. It revealed weak FTIR absorptions at 807-590 cm^{-1}, because glycosidic bond cleavage increased C-O/O-H moieties, confirming chain conformation and surface morphology modifications[10]. Frequency-dependent ultrasound effects on polysaccharide structures correlate strongly with acoustic field characteristics. Moreover, the arc-shaped flat-plate ultrasound significantly enhanced the solubility of xanthan gum with marked viscosity changes demonstrating superior modification efficacy. However, frequency-mode interactions remained unexamined, warranting systematic

investigation of frequency combinations in this novel ultrasonic transducer system.

Therefore, this study focuses on the frequency modes generated by the arc-shaped flat-plate transducer, investigating their propagation characteristics within xanthan gum viscous systems and their subsequent impacts on the rheological properties of the medium.

2.2. Effects of Multi-Frequency Arc-Shaped Flat-Plate Ultrasound on Solubility and Viscosity of Xanthan Gum

In the viscous system of 4.5 $g \cdot L^{-1}$ xanthan gum, effects of multi-frequency modes of arc-shaped flat-plate ultrasound on the solubility (A) and viscosity (B) are illustrated in Figure S2. Generally, all modified samples showed an increasing trend in solubility and a decreasing trend in viscosity. However, different frequency modes exhibited distinct effects on the solubility and viscosity. Specifically, the 68 kHz mono-frequency ultrasound showed the poorest solubility (60.0%), which may be attributed to the weaker penetration capability of higher-frequency ultrasonic waves. The 20-40 kHz dual-frequency ultrasound achieved the highest solubility improvement (85.6%), representing a 1.38-fold enhancement.

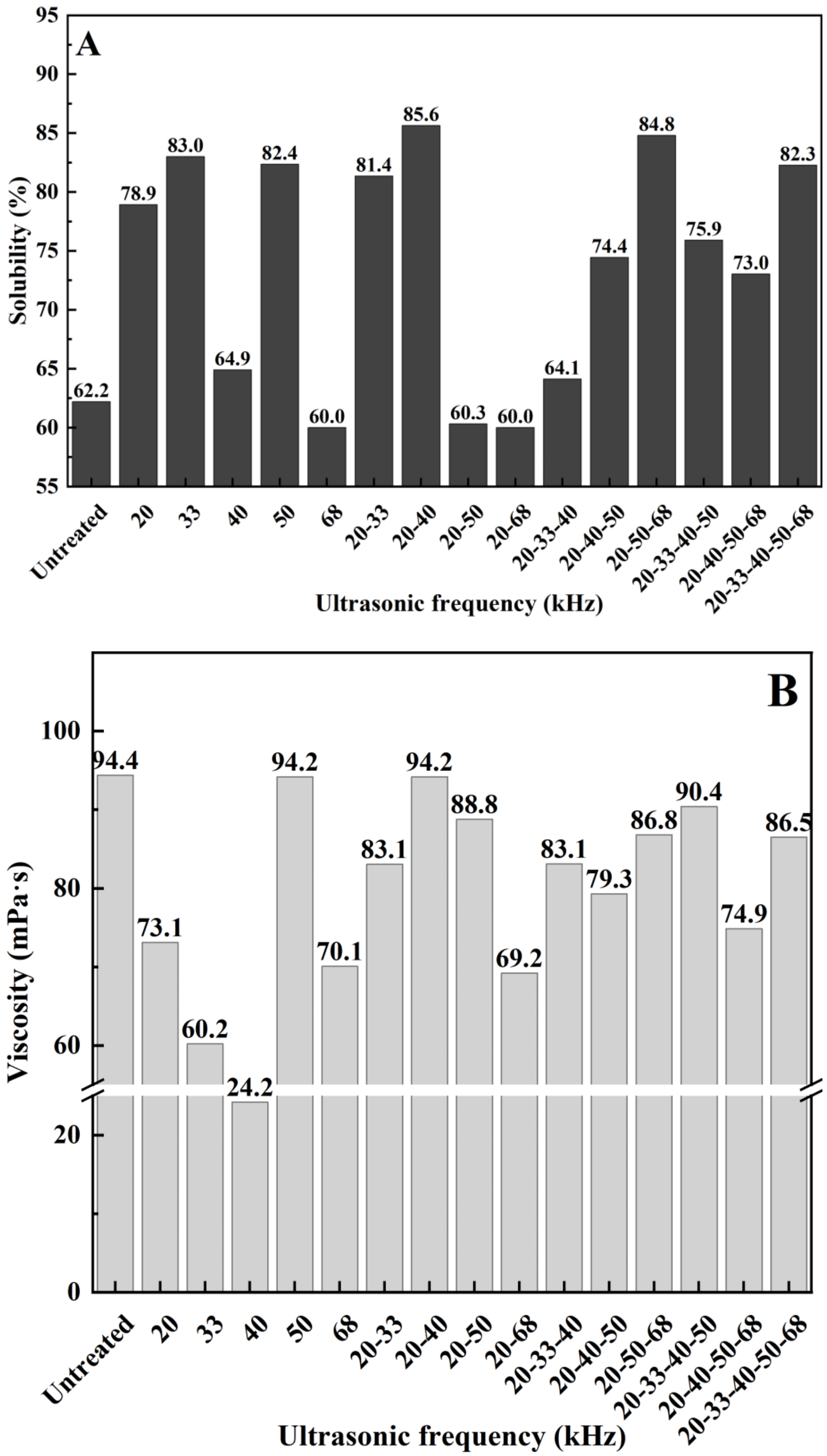

A
Solubility (%)
95
90
85
80
75
70
65
60
55
62.2
78.9
83.0
64.9
82.4
60.0
81.4
85.6
60.3
60.0
64.1
74.4
84.8
75.9
73.0
82.3
Untreated
20
33
40
50
68
20-33
20-40
20-50
20-68
20-33-40
20-40-50
20-50-68
20-33-40-50
20-40-50-68
20-33-40-50-68
Ultrasonic frequency (kHz)
B
Viscosity (mPa·s)
100
80
60
20
0
94.4
73.1
60.2
24.2
94.2
70.1
83.1
94.2
88.8
69.2
83.1
79.3
86.8
90.4
74.9
86.5
Untreated
20
33
40
50
68
20-33
20-40
20-50
20-68
20-33-40
20-40-50
20-50-68
20-33-40-50
20-40-50-68
20-33-40-50-68
Ultrasonic frequency (kHz)

Figure S2. Effects of arc-shaped flat-plate ultrasound with different frequency modes on XG solution (4.5 g·L^{-1}). (A) Solubility, (B) viscosity. (Note: Data for 40 kHz cited from **Figure S1** of Supplementary Materials).

Comparative analysis of various ultrasonic frequency modes showed that the 33 kHz mono-frequency, 20-40 kHz dual-frequency, and 20-50-68 kHz triple-frequency modes exhibited the most significant improvement in the solubility of xanthan gum. Correspondingly, under the 33 kHz mono-frequency mode, the viscosity decreased to 60.2 mPa·s, representing an approximately 1.5-fold reduction, while the 20-40 kHz dual-frequency and 20-50-68 kHz triple-frequency modes demonstrated relatively minor effects on viscosity alteration.

With MW exceeding 1000 Da, xanthan gum was classified as a high-molecular-weight substance, where the solubility and viscosity were not directly correlated, and enhanced solubility contributed to improved thickening properties, which were associated with intermolecular chain interactions[11]. Furthermore, mono-frequency ultrasound typically operates at specific frequencies, enabling targeted modification of pectin, such as viscosity reduction[12]. In contrast, multi-frequency ultrasound employs multiple simultaneous frequencies to generate more complex vibrational and thermal effects, thereby modulating pectin's physical and chemical properties across a broader spectrum[13].

As an efficient non-thermal processing technology, ultrasonic treatment primarily functions through cavitation-induced mechanical, chemical, and thermal effects that alter molecular structures and solution properties. Meanwhile, varying liquid environments differentially affected acoustic wave propagation, thereby directly influencing modification outcomes[14,15]. Ultrasonic frequencies exhibited significant variations in treatment efficacy, as evidenced by researchers who used 20 kHz mono-

frequency ultrasound to treat soy protein emulsions, resulting in protein structure unfolding, high encapsulation efficiency, and enhanced thermal stability[16]. Multi-frequency ultrasound (*e.g.*, 22-33 kHz combination) achieved a 59% maximum conversion rate of olive oil into structured lipids within merely 4 h[17]. This was because dual-frequency synergy induced bubbles to generate harmonic, subharmonic, ultraharmonic waves and other frequencies, creating additional oscillations that established heterogeneous acoustic pressure fields, thereby increasing cavitation bubble populations and intensifying cavitation effects[18]. Furthermore, multi-frequency combinations enhanced acoustic cavitation effects, promoting non-covalent interactions between proteins and polysaccharides[19]. Collectively, these findings demonstrate that ultrasound-induced cavitation effects can substantially modify molecular architectures and solution characteristics through mechanical, chemical and thermal actions. A key consideration is that only optimized ultrasonic frequencies could induce molecular resonance in xanthan gum, although viscous systems exhibited ultrasound attenuation effects. To elucidate the mechanism of arc-shaped flat-plate ultrasound action, time–frequency domain monitoring of discrete propagation points was employed to systematically analyze its propagation behavior in viscous systems of xanthan gum.

2.3. Time-Domain Acoustic Field Monitoring of Arc-Shaped Flat-Plate Ultrasound in Xanthan Gum Viscous Systems

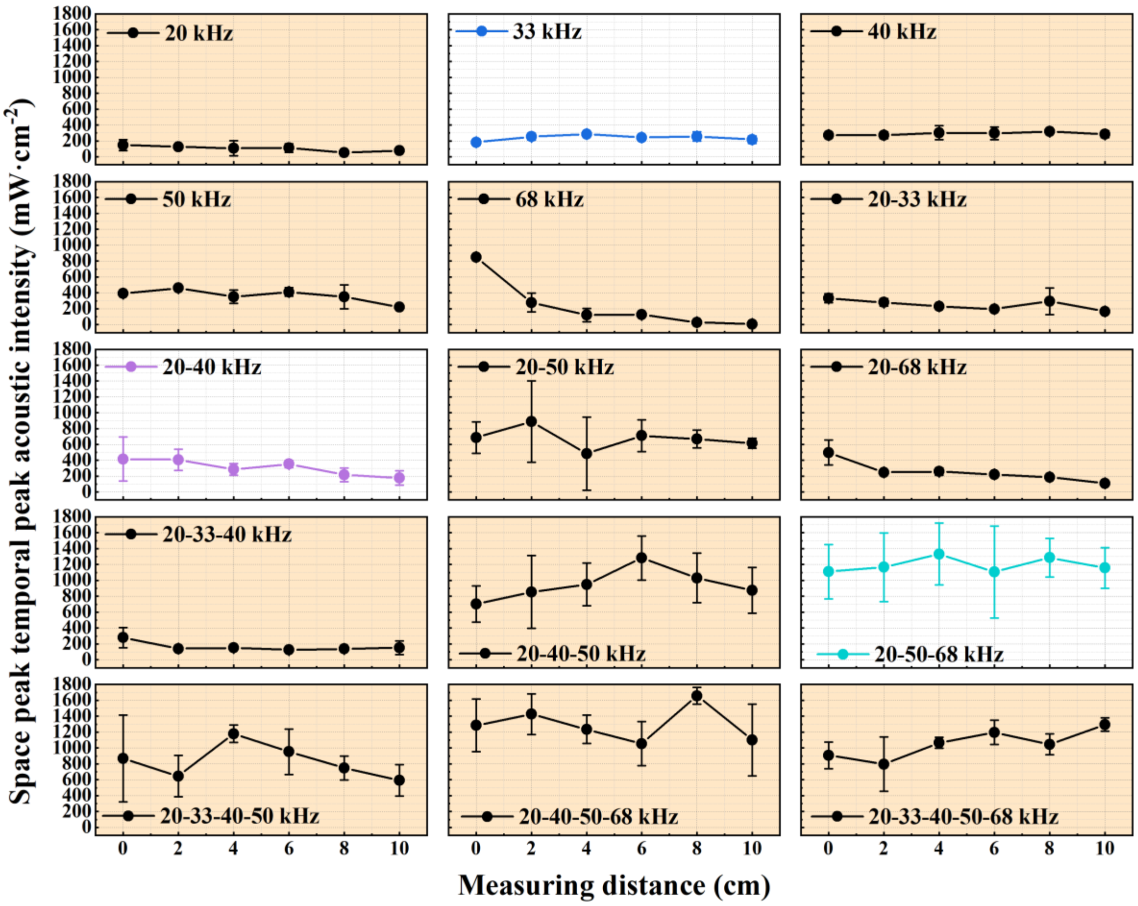

Figure S3. Space peak temporal peak acoustic intensity (I_{SPTP}) distribution monitored at discrete points in viscous system of XG (4.5 g·L^{-1}) under 15 frequency modes of arc-shaped flat-plate ultrasound (Note: Data for 4.5 g·L^{-1} cited from **Figure S2**).

Figure S3 demonstrates the monitoring of I_{SPTP} at discrete points using PVDF sensors in the viscous system of 4.5 g·L^{-1} xanthan gum under 15 frequency modes of arc-shaped flat-plate ultrasound, investigating the propagation characteristics of different ultrasonic frequency modes. The ultrasonic frequency modes significantly influenced the acoustic intensity distribution characteristics. Specifically, the I_{SPTP} of the ultrasonic field followed this order: penta-frequency>tetra-frequency>tri-frequency>dual-frequency>mono-frequency. Moreover, high frequencies in mono-frequency modes exhibited more pronounced ultrasonic attenuation. For instance, the 68 kHz high-frequency mode showed a high I_{SPTP} near the transducer working surface

but underwent rapid attenuation with increasing propagation distances. Correlating these findings with modification results in Figure S2, modes (33, 20-40, 20-50-68 kHz), significantly enhanced the solubility, were characterized by more uniform I_{SPTP} distributions and consistent spatial energy dispersion, indicating effective penetration. Although the 20-50 kHz dual-frequency mode achieved high I_{SPTP}, its substantial variability suggested that only optimized frequency combinations could generate superior mechanical agitation and cavitation yield[20]. Furthermore, the tri-frequency 20-50-68 kHz ultrasound combination maintained high I_{SPTP} at propagation distances ≥6 cm, with the solubility increasing to 84.8%, suggesting a "combined resonance" effect in multi-frequency ultrasound that generated a broader bubble size distribution[21]. Regarding propagation distance, both 68 kHz mono-frequency and 20-50-68 kHz tri-frequency modes achieved I_{SPTP} values of about 1100 $mW·cm^{-2}$ at the nearest position (0 cm) to the ultrasonic transducer surface. However, between 2 and 6 cm, the 68 kHz mono-frequency ultrasound showed significant reduction in I_{SPTP}, while the 20-50-68 kHz tri-frequency ultrasound maintained relatively stable values. At 8-10 cm distances, substantial fluctuations in I_{SPTP} occurred with notable ultrasonic attenuation, attributable to consistent energy dissipation mechanisms during ultrasound–medium interactions, where shorter wavelengths of high-frequency ultrasound enhanced medium absorption and thermal conversion, accelerating acoustic intensity decay. Multi-frequency ultrasound enabled more extensive energy dissipation[22].

In summary, based on a comprehensive evaluation of ultrasonic attenuation characteristics, energy distribution uniformity, and modification efficacy, three frequency modes (33, 20-40, 20-50-68 kHz) were selected for an in-depth analysis of

the time–frequency domain characteristics to elucidate their synergistic enhancement mechanisms.

2.4. Viscoelastic Property

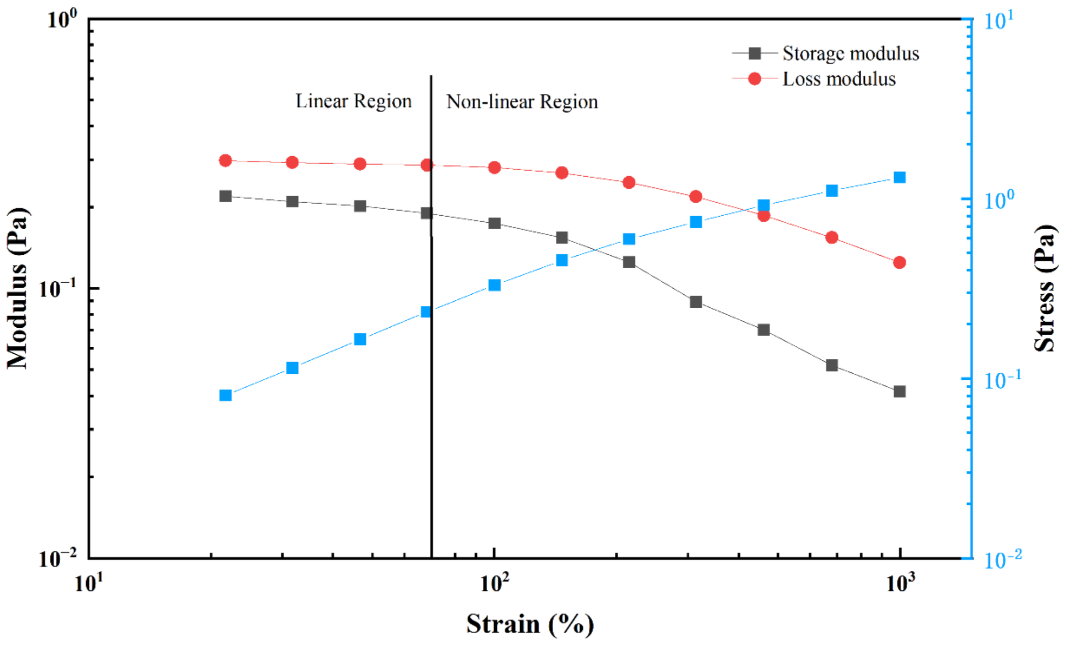

Figure S4. Strain scan chart for 4.5 g·L^{-1} XG.

For many polysaccharides, the linear viscoelastic region typically remains within a relatively low strain range. However, for the specific polymer solution system studied in this study, our strain scan results clearly indicate that, as shown in Figure S4, taking a 4.5 g·L^{-1} xanthan gum solution as an example, its linear viscoelastic region extends to about 60% strain. Therefore, the selected 50% strain amplitude still falls within the linear region. This choice enables us to obtain stronger rheological signals while ensuring the structural integrity of the material is not affected, thereby improving the quality of frequency scanning data.

References

1. Zeng, Q.; Zhang, L.; Liao, W.; Liu, J.; Yuan, F.; Gao, Y. Effect of xanthan gum co-extruded with OSA starch on its solubility and rheological properties. *LWT* **2021**, *147*, 111588, doi:https://doi.org/10.1016/j.lwt.2021.111588.
2. Su, Y.; Chen, Y.; Zhang, L.; Adhikari, B.; Xu, B.; Li, J.; Zheng, T. Synthesis and characterization of lotus seed protein-based curcumin microcapsules with enhanced solubility, stability, and sustained release. *J Sci Food Agric* **2022**, *102*, 2220-2231, doi:10.1002/jsfa.11560.
3. Cardoso de Castro, C.S.; Santo Filho, D.M.d.E.; Siqueira, J.Renato R.; Barbosa, A.P.F.; Rodrigues, C.R.d.C.; Cabral, M.L.; da Silva, E.M.; Baldner, F.d.O.; Gouveia, J.M.G. Evaluation of the metrological performance of two kinds of rotational viscometers by means of viscosity reference materials. *Journal of Petroleum Science and Engineering* **2016**, *138*, 292-297, doi:https://doi.org/10.1016/j.petrol.2015.12.003.
4. Azari, S.R.; Hojjatoleslamy, M.; Mousavi, Z.E.; Kiani, H.; Jalali, S.M.A. Investigating the impact of sodium alginate and xanthan hydrocolloids on the rheological Doogh properties (Iranian dairy drink). *Food Hydrocolloids for Health* **2025**, *7*, 100206, doi:https://doi.org/10.1016/j.fhfh.2025.100206.
5. Zhong, W.; Yu, Y.; Zhang, B.; Tao, D.; Fang, J.; Ma, F. Effect of H2O2-assisted ultrasonic bath on the degradation and physicochemical properties of pectin. *International Journal of Biological Macromolecules* **2024**, *258*, 128863, doi:https://doi.org/10.1016/j.ijbiomac.2023.128863.
6. García-Ochoa, F.; Santos, V.E.; Casas, J.A.; Gómez, E. Xanthan gum: production, recovery, and properties. *Biotechnology Advances* **2000**, *18*, 549-579, doi:https://doi.org/10.1016/S0734-9750(00)00050-1.
7. Shatwell, K.P.; Sutherland, I.W.; Ross-Murphy, S.B. Influence of acetyl and pyruvate substituents on the solution properties of xanthan polysaccharide. *International Journal of Biological Macromolecules* **1990**, *12*, 71-78, doi:https://doi.org/10.1016/0141-8130(90)90056-G.
8. Jia, Y.; Li, Q.; Jiang, F.; Huang, X.; Zeng, L.; Zhang, Y.; Xu, L. Ultrasonic degradation of mulberry twigs polysaccharides: Effect on in vitro hypoglycemic activity and prebiotic potential. *International Journal of Biological Macromolecules* **2025**, *310*, 143356, doi:https://doi.org/10.1016/j.ijbiomac.2025.143356.
9. Mansour, M.; Khoder, R.M.; Xiang, L.; Zhang, L.L.; Taha, A.; Yahya, A.; Wu, T.; Barakat, H.; Khalifa, I.; Xiaoyun, X. Effect of ultrasonic degradation on the physicochemical property, structure characterization, and bioactivity of Houttuynia cordata polysaccharide. *Ultrasonics Sonochemistry* **2025**, *116*, 107331, doi:https://doi.org/10.1016/j.ultsonch.2025.107331.
10. Liu, W.; Qin, Y.-M.; Shi, J.-Y.; Wu, D.-L.; Liu, C.-Y.; Liang, J.; Xie, S.-Z. Effect of ultrasonic degradation on the physicochemical characteristics, GLP-1 secretion, and antioxidant capacity of Polygonatum cyrtonema polysaccharide. *International Journal of Biological Macromolecules* **2024**, *274*, 133434, doi:https://doi.org/10.1016/j.ijbiomac.2024.133434.
11. Nsengiyumva, E.M.; Alexandridis, P. Xanthan gum in aqueous solutions: Fundamentals and applications. *International Journal of Biological Macromolecules* **2022**, *216*, 583-604, doi:https://doi.org/10.1016/j.ijbiomac.2022.06.189.
12. Zhang, J.; Tao, L.; Yang, S.; Li, Y.; Wu, Q.; Song, S.; Yu, L. Water absorption behavior of starch: A review of its determination methods, influencing factors, directional modification, and food

applications. *Trends in Food Science & Technology* **2024**, *144*, 104321, doi:https://doi.org/10.1016/j.tifs.2023.104321.
13. Ahmed, Z.; Xu, B.; Farooq, U.; Manzoor, M.F.; Awad, M.F.; Ashraf, J.; Tufail, T.; Abdi, G. Impact of multi-frequency ultrasound processing with different treatment times on the structural quality of frozen wheat dough. *Ultrasonics Sonochemistry* **2024**, *111*, 107116, doi:https://doi.org/10.1016/j.ultsonch.2024.107116.
14. Ranasinghe, M.; Stathopoulos, C.; Sundarakani, B.; Maqsood, S. Valorizing date seeds through ultrasonication to enhance quality attributes of dough and biscuit, Part-1: Effects on dough rheology and physical properties of biscuits. *Ultrasonics Sonochemistry* **2024**, *109*, 107015, doi:https://doi.org/10.1016/j.ultsonch.2024.107015.
15. Kang, J.; Yue, H.; Li, X.; He, C.; Li, Q.; Cheng, L.; Zhang, J.; Liu, Y.; Wang, S.; Guo, Q. Structural, rheological and functional properties of ultrasonic treated xanthan gums. *International Journal of Biological Macromolecules* **2023**, *246*, 125650, doi:10.1016/j.ijbiomac.2023.125650.
16. Zhu, J.; Wang, H.; Miao, L.; Chen, N.; Zhang, Q.; Wang, Z.; Xie, F.; Qi, B.; Jiang, L. Curcumin-loaded oil body emulsions prepared by an ultrasonic and pH-driven method: Fundamental properties, stability, and digestion characteristics. *Ultrasonics Sonochemistry* **2023**, *101*, 106711, doi:https://doi.org/10.1016/j.ultsonch.2023.106711.
17. Khasgiwale, V.N.; Waghmare, J.T.; Gogate, P.R. Intensified synthesis of olive oil-based structured lipids based on enzymatic acidolysis using multi-frequency ultrasound. *Chemical Engineering Science* **2025**, *302*, 120947, doi:https://doi.org/10.1016/j.ces.2024.120947.
18. Zeng, L.; Huang, C.; Tang, Y.; Wang, C.; Lin, S. Tetracycline degradation by dual-frequency ultrasound combined with peroxymonosulfate. *Ultrasonics Sonochemistry* **2024**, *106*, 106886, doi:https://doi.org/10.1016/j.ultsonch.2024.106886.
19. Qayum, A.; Rashid, A.; Liang, Q.; Kang, L.; Ahmed, Z.; Hussain, M.; Virk, M.S.; Ekumah, J.-N.; Ren, X.; Ma, H.; et al. Multi-scale ultrasound induced composite coacervates of whey protein and pullulan polysaccharide on emulsion forming and stabilizing mechanisms. *Colloids and Surfaces B: Biointerfaces* **2024**, *234*, 113709, doi:https://doi.org/10.1016/j.colsurfb.2023.113709.
20. Yang, X.; Bian, C.; Dong, Y.; Xie, J.; Mei, J. Effects of different power multi-frequency ultrasound-assisted thawing on the quality characteristics and protein stability of large yellow croaker (Larimichthys crocea). *Food Chemistry: X* **2024**, *23*, 101559, doi:https://doi.org/10.1016/j.fochx.2024.101559.
21. Yan, W.; Chen, Z.; Zhang, C.; Xu, Y.; Han, C.; Yue, L.; Kong, Q.; Zheng, Q.; Tian, W.; Xu, B. Multi-frequency power ultrasound (MFPU) pretreatment of crayfish (Procambarus clarkii): Effect on the enzymatic hydrolysis process and subsequent Maillard reaction. *Ultrasonics Sonochemistry* **2024**, *111*, 107140, doi:https://doi.org/10.1016/j.ultsonch.2024.107140.
22. Ma, H.; Huang, L.; Peng, L.; Wang, Z.; Yang, Q. Pretreatment of garlic powder using sweep frequency ultrasound and single frequency countercurrent ultrasound: Optimization and comparison for ACE inhibitory activities. *Ultrasonics Sonochemistry* **2015**, *23*, 109-115, doi:https://doi.org/10.1016/j.ultsonch.2014.10.020.
